# Effects of continuous cropping on yield, medicinal quality, and rhizosphere soil microecological environment of *Atractylodes chinensis* in the Horqin Sandy land

**DOI:** 10.3389/fmicb.2026.1943837

**Published:** 2026-10-27

**Authors:** Kaiqi Wang, Kai Li, Dezhi Sun, Yuan Guo, Zhijun Li, Yanqin Zhao, Yifan Yin, Junying Jia

**Affiliations:** 1 College of Agronomy, Inner Mongolia Minzu University, Tongliao, Inner Mongolia, China; 2 Ulanqab City Agricultural Technology Extension Center, Ulanqab, Inner Mongolia, China; 3 College of Prataculture, Inner Mongolia Minzu University, Tongliao, Inner Mongolia, China

**Keywords:** *Atractylodes chinensis*, continuous cropping obstacles, microbial community, rhizospheric soil, soil chemical properties

## Abstract

**Background:**

Continuous cropping is a major constraint that limits the sustainable cultivation of medicinal plants. However, the effects of continuous cropping on yield, medicinal quality, and the rhizosphere soil microecological environment of *Atractylodes chinensis*, particularly in the Horqin Sandy Land, remain unclear. In this study, we conducted a field experiment with continuous cropping and rotation systems to compare the yield, bioactive compound content, soil chemical properties, and rhizosphere microbial communities of *A. chinensis* and to further elucidate their potential relationships.

**Methods:**

Plant and soil samples were collected to assess plant yield and bioactive compounds, soil chemical properties, and soil microbial communities using high-throughput sequencing, followed by bioinformatic analysis of the sequencing data.

**Results:**

Compared with crop rotation, continuous cropping significantly reduced yield and the contents of major bioactive compounds, including atractylodin, atractylon and β-eudesmol. Continuous cropping significantly increased soil pH, electrical conductivity (EC), and available potassium, and decreased alkali-hydrolyzable nitrogen and organic matter (OM). Additionally, continuous cropping decreased fungal richness (Chao1 index) and increased bacterial diversity (Shannon index) and evenness (Pielou’s evenness). *Fusarium* and *Volutella* were significantly enriched, whereas *Nocardioides* and *Arthrobacter* were depleted. The predicted microbial functional potential associated with carbon and nitrogen cycling significantly decreased under continuous cropping. The correlation analysis revealed that the yield of *A. chinensis* was negatively correlated with soil EC and positively correlated with OM content, and the relative abundances of *Heydenia* and *Enterocarpus*. Atractylon content was positively correlated with *Aleuria* and *Acremonium* and negatively correlated with *Volutella*.

**Conclusion:**

These findings demonstrate that continuous cropping reduces the yield and medicinal quality of *A. chinensis* in the Horqin Sandy Land and is associated with the deterioration of several soil chemical properties and dysbiosis of the rhizosphere microbial community. This study provides novel insights into the continuous cropping constraint associated with *A. chinensis* and its ecological consequences in the Horqin Sandy Land and establishes a theoretical foundation for optimizing cropping systems, maintaining soil ecological health, and improving the yield and quality of this medicinal herb.

## Highlights

- Continuous cropping reduced both yield and bioactive compound accumulation in *A. chinensis*.
- Continuous cropping altered the relative abundances of specific microbial taxa in the rhizosphere of *A. chinensis*.
- Several soil chemical properties and microbial biomarkers were associated with *A. chinensis* yield and bioactive compound accumulation.

## 1 Introduction

*Atractylodes chinensis* (DC.) Koidz. is a perennial medicinal herb belonging to the Asteraceae family, and its dried rhizome is widely used in traditional Chinese medicine. The major bioactive constituents of *A. chinensis*, including atractylodin, atractylon, and β-eudesmol, exhibit various pharmacological activities and contribute to its broad clinical applications in China (National Pharmacopoeia Committee, 2020; Wang, 2023). Recently, the increasing domestic and international demand for *A. chinensis* has substantially depleted its wild resources, making artificial cultivation the primary source of commercial production (Xiao et al., 2025). However, intensified regulations aimed at preventing the conversion of arable land from grain to non-grain production have restricted the expansion of cultivation areas [General Office of the State Council Opinions of the General Office of the State Council on Preventing the "non-grain" of Cultivated land and Stabilizing Grain Production (State Office. No.44), 2020]. Consequently, the repeated cultivation of *A. chinensis* on the same land has become increasingly prevalent, resulting in severe continuous cropping obstacles (CCOs). These challenges are characterized by suppressed plant growth and development, reduced yield, and the deterioration of medicinal quality (Haq et al., 2025), thereby threatening the sustainable development of the *A. chinensis* industry and negatively affecting regional economies and farmers’ incomes.

CCOs are a series of agroecological challenges that occur when the same or closely related plant species are repeatedly cultivated on the same land over extended periods under conventional agricultural practices, thereby resulting in substantial reductions in crop yield and quality (Chen et al., 2022). The underlying mechanisms are highly complex and involve intricate interactions within the soil–plant–microbiome continuum. Continuous cropping substantially influences soil physicochemical properties, enzyme activities, root exudation patterns, and microbial community composition, collectively disrupting soil ecosystem functions. Continuous cropping degrades soil physicochemical properties, as evidenced by reduced soil permeability, nutrient imbalances, and increased electrical conductivity (Liu, 2021). Long-term continuous cropping suppresses the activity of key soil enzymes, including urease, sucrase, and acid phosphatase (Lin et al., 2025), thereby impairing nutrient cycling and reducing the availability and utilization efficiency of essential nutrients by plants (Liu et al., 2022). Additionally, the accumulation of root exudates is another key contributor to CCO (Bennett et al., 2012; Berendsen et al., 2012). Root exudates contain various secondary metabolites and allelochemicals that can recruit pathogenic microorganisms or stimulate their proliferation and metabolic activities, thereby altering the rhizosphere microbial community composition and disrupting the ecological balance of native microbial communities (Zhang et al., 2019; Zhu et al., 2020). Furthermore, microorganisms involved in nutrient cycling and degradation of harmful allelochemicals decrease under long-term continuous cropping (Zhang et al., 2020). The interactions among these factors progressively deteriorate the soil microenvironment and initiate a self-reinforcing negative feedback loop, thereby impairing nutrient acquisition, reducing plant resilience to environmental stress, and adversely affecting plant growth, development, and accumulation of pharmacologically active compounds (Gu et al., 2023).

Previous studies on *A. chinensis* have predominantly focused on its bioactive components, cultivation techniques, and disease management. The rhizosphere soil microbial communities of *A. chinensis* have recently attracted increasing attention; however, existing studies have mainly examined the differences in rhizosphere microbial communities among *A. chinensis* from different regions and their association with medicinal quality (Bai et al., 2025; Lu et al., 2026). In contrast, systematic investigations into the continuous cropping of *A. chinensis* remain limited, particularly in the Horqin Sandy Land, a unique and ecologically fragile agricultural ecosystem in northern China. The effects of continuous cropping on the yield and quality of *A. chinensis* and its soil microecological environment remain to be comprehensively elucidated. Therefore, this study used a continuous cropping system of *A. chinensis* in Horqin Sandy Land as a model to comprehensively evaluate plant yield and quality, soil chemical properties, and rhizosphere microbial communities following continuous cropping. Specifically, we aimed to identify the changes in soil chemical properties, microbial community composition, and predicted microbial functional potential induced by continuous cropping and to explore the associations between these changes and the yield and major bioactive compounds of *A. chinensis*. By integrating these responses, this study provides a crop- and region-specific perspective on the ecological consequences of continuous cropping on medicinal plants and identifies potential microbial taxa and soil indicators associated with changes in yield and medicinal quality, thereby providing a theoretical foundation for mitigating CCO and improving the yield and medicinal quality of *A. chinensis* in the Horqin Sandy Land.

## 2 Materials and methods

### 2.1 Experimental design

The field experiment was conducted at the Chinese (Mongolian) Medicinal Plant Research Base in Naiman Banner, located in the central Horqin Sandy Land, China (42° 14′17″N, 120° 20′35″E). The study area is characterized by a temperate continental monsoon climate, with a mean annual temperature of 6.0–6.5 °C, an average annual precipitation of 290–350 mm, and an average frost-free period of approximately 150 days. Before the experiment, maize was planted continuously throughout the experimental field for several years. The field experiments were initiated in 2020. The experimental sites were divided into six plots, each measuring 60 m^2^ (6 × 10 m). Before planting, baseline soil samples were collected from each plot using a five-point sampling method. Soil from the five sampling points within each plot was thoroughly mixed to obtain a composite sample. Consequently, six composite soil samples were collected and used to determine the baseline soil physicochemical properties. Two cropping systems were established, continuous cropping (T1) and crop rotation (T2), with three randomly assigned plots serving as replicates for each treatment. Each plot was considered an independent experimental unit. The T1 plots were continuously planted with *A. chinensis* for 3 consecutive years (one cropping cycle) until harvest, whereas the T2 plots were planted with *Astragalus membranaceus* for 3 consecutive years. Following harvesting, all plots were uniformly plowed and prepared into level beds. Uniformly growing 2-year-old *A. chinensis* seedlings, provided by Naiman Banner Guoan Agricultural Development Co., Ltd., were transplanted into all plots on May 2, 2023, at a spacing of 20 × 40 cm, corresponding to a planting density of 125,000 plants/hm^2^. Samples were collected from each plot 1 year after transplantation for subsequent analyses. Field management practices were maintained consistently across the preceding crops (*A. chinensis* and *A. membranaceus*) and experimental *A. chinensis* plants. In early spring, an abamectin insecticide (1.8% abamectin, EC; Stanley, China) was applied in a single application to control aphids at a rate of 195 mL/hm^2^. Irrigation was provided as needed throughout the growing season based on soil moisture and precipitation, and weeds were manually removed at regular intervals. No chemical fertilizers, herbicides, fungicides, or organic amendments were used during the experimental period.

### 2.2 Plant and soil sample collection

Sampling was conducted at the following four developmental stages: late leaf expansion (June 27), flowering (July 27), fruiting (August 27), and harvesting (September 27). At each sampling point, 12 uniformly growing *A. chinensis* plants were randomly selected from each plot as subsamples and pooled to generate a single composite sample per plot. Three composite samples were obtained per treatment at each sampling time point, each derived from one of the three independent plots, thereby constituting three independent biological replicates. Following collection, organs were separated immediately; rhizomes were stored at −20 °C, oven-dried and ground for subsequent analysis of bioactive compounds. Rhizosphere soil was collected concurrently by brushing the soil that adhered to the roots of *A. chinensis*. After thorough mixing, fresh soil samples were divided into two portions: one was air-dried for the determination of soil chemical properties; the other was immediately flash-frozen in liquid nitrogen following collection, transferred to a − 80 °C freezer (Lauber et al., 2010) within 2 h, and processed for microbial sequencing within 3 months.

### 2.3 Determination of yield and bioactive compounds

Yield was determined at the harvest stage (September 27) using a plot-based sampling approach. Three replicate plots were established for each treatment, and a 10 m^2^ sampling quadrat was randomly positioned within each replicate plot. All *A. chinensis* plants within each 10 m^2^ sampling quadrat were excavated and collected. The rhizomes (crude medicinal material) were rinsed, surface water was blotted off, and fresh weight was recorded. Rhizomes were subsequently dried to constant weight in a 60 °C oven; the dry weight was recorded, and the actual moisture content of the sample was calculated as follows: (fresh weight–dry weight)/fresh weight. The dry weight of each 10 m^2^ sampling quadrat was recalibrated to a standard moisture content of 13% (w/w, wet basis) to obtain the standardized weight of the crude medicinal material per quadrat, which was expressed on a dry weight basis standardized to 13% moisture content. The per-hectare yield of crude medicinal material was calculated as follows:

Y=Wd×1,000

Where Y is the crude drug yield (kg/hm^2^), Wd is the mean standardized dry weight of the crude drug per 10 m^2^ of the sampling area (kg), and 1,000 is the conversion factor from a 10 m^2^ sampling area to 1 hm^2^ (10,000 m^2^/10 m^2^ = 1,000).

Three additional *A. chinensis* plants were randomly selected from each plot, separated from those excavated for yield determination. The roots, stems, and leaves of each plant were separated, and the rhizomes were rinsed thoroughly. The rhizomes were fixed at 105 °C for 10 min to rapidly inactivate endogenous enzymes before drying, to prevent enzymatic degradation of bioactive compounds during the subsequent drying process (Huang et al., 2023) and dried to a constant weight in a 60 °C oven. The dried rhizomes were pulverized and passed through a No. 3 sieve. An accurately weighed 0.2 g portion of the powder was placed in a glass-stoppered conical flask, 25 mL of methanol was precisely added, and the total mass was determined. The mixture was extracted via ultrasonication for 30 min at 300 W and 40 kHz, removed, allowed to cool to room temperature, and the mass was subsequently redetermined. Any weight loss was corrected for through methanol supplementation. Following thorough mixing, the solution was filtered through a 0.45 μm organic microporous membrane, and the successive filtrate was collected as the test solution. Each sample was prepared in triplicate. Quantification was performed on an LC-2030 high-performance liquid chromatography system equipped with an Agilent Zorbax SB-C18 column (4.6 mm × 250 mm, 5 μm) maintained at 30 °C. The mobile phase consisted of acetonitrile (A) and 0.1% aqueous phosphoric acid (B), delivered at a flow rate of 0.6 mL/min with the following gradient elution program: 0–10 min, 30% A; 10–12 min, 30% A; 12–20 min, 30% → 70% A; 20–22 min, 70% A; 22–30 min, 70% → 80% A; 30–32 min, 80% A; 32–36 min, 80% → 30% A; and 36–40 min, 30% A. The injection volume was 5 μL, and the detection wavelength was uniformly set at 203 nm, with β-eudesmol and atractylodin monitored at 0–19 min and atractylon at 19–25 min. The reference standard was atractylodin (batch no. LB465178, purity ≥ 98%), β-eudesmol (batch no. JB240678, purity ≥ 95%), and atractylon (batch no. LB485919, purity ≥ 95%), purchased from Shanghai Yuanye Biotechnology Co., Ltd. Acetonitrile was of chromatographic grade, phosphoric acid was of analytical grade, and ultrapure water was used for all experiments. The method validation demonstrated good linearity for the three active compounds over their respective concentration ranges. Using the mass concentration of the reference standards (x, mg/g) as the abscissa and the corresponding peak area (Y) as the ordinate, the regression equations were as follows: atractylodin, Y = 1913.3x + 263.77 (R^2^ = 0.9993); atractylon, Y = 216.86x + 72.854 (R^2^ = 0.9993); and β-eudesmol, Y = 117.06x + 2.5927 (R^2^ = 0.9999). The calibration ranges corresponded to sample contents of 0.16–15.75, 0.28–27.53, and 1.69–169.36 mg/g for atractylodin, atractylon, and β-eudesmol, respectively, thereby covering the measured contents of all test samples. Instrument precision was satisfactory: when the same mixed reference solution was injected six consecutive times, the relative standard deviations (RSDs) of the peak areas of atractylodin, atractylon, and β-eudesmol were 0.46%, 0.88%, and 0.31%, respectively, all <2%. The test solutions remained stable within 24 h, with RSDs of the peak areas of atractylodin, atractylon, and β-eudesmol of 0.54%, 0.81%, and 0.82%, respectively, all <2%. The accuracy of the method was verified using a spiked recovery test, and the mean recoveries of the three compounds ranged from 96.0 to 101.4%, satisfying the accuracy requirements for the quantitative determination of active compounds in medicinal plants. Converted based on a sample mass of 0.2 g and a final volume of 25 mL, the limits of detection for atractylodin, atractylon, and β-eudesmol were 0.0008, 0.0048, and 0.0213 mg/g, respectively, and the corresponding limits of quantification (LOQs) were 0.0021, 0.0138, and 0.0650 mg/g. The measured contents of the three active compounds in the samples were all more than 10 times higher than their respective LOQs, indicating that the sensitivity of the method completely satisfied the determination requirements of the samples in this study.

### 2.4 Determination of soil chemical properties

Soil pH was measured using a pH meter at a soil: water ratio of 1:5 (w/v; Zhao J. et al., 2022). Soil electrical conductivity (EC) was determined using a DDS-307 conductivity meter (Shanghai INASE Scientific Instrument Co., Ltd., China) at a soil-to-water ratio of 1:2.5 (w/v; Peng et al., 2014). Soil alkali-hydrolyzable nitrogen (AN) was determined using the alkali-hydrolytic diffusion technique (Bai et al., 2026). Available phosphorus (AP) was extracted with NaHCO₃ and quantified using the molybdenum-antimony contrast method (Du et al., 2026). Available potassium (AK) was extracted with NH₄OAc and measured using a flame photometer (Zhang et al., 2026). Soil OM was determined using the potassium dichromate oxidation method with external heating (Lu, 2000).

### 2.5 Soil microbial high-throughput sequencing

Rhizosphere Rhizosphere soil samples collected at the harvest stage and stored at −80 °C were used for microbial community analysis. DNA extraction, PCR amplification, and sequencing were performed by LC-Bio Technologies Co., Ltd. (Hangzhou, China). The total microbial genomic DNA was extracted using a modified cetyltrimethylammonium bromide method. The extracted DNA was quantified using a Qubit fluorometer (Invitrogen, USA) and stored at −20 °C until further analysis. For bacterial community analysis, the V3–V4 region of the 16S rRNA gene was amplified using the primer pair 341F (5′-CCTACGGGNGGCWGCAG-3′) and 805R (5′-GACTACHVGGGTATCTAATCC-3′) (Herlemann et al., 2011; Klindworth et al., 2013). For fungal community analysis, the ITS2 region was amplified using the primer pair fITS7F (5′-GTGARTCATCGAATCTTTG-3′) and ITS4R (5′-TCCTCCGCTTATTGATATGC-3′; Karlsson et al., 2014). The PCR was performed in a total reaction volume of 25 μL: DNA template 50 ng, forward primer (1 μM) 2.5 μL, reverse primer (1 μM) 2.5 μL, Pusion Hot Start Flex 2X Master Mix 12.5 μL, and finally ddH_2_O up to 25 μL. PCR conditions consisted of initial denaturation at 98 °C for 30 s, followed by 32 cycles of denaturation (98 °C, 10 s), annealing (54 °C, 30 s), and extension (72 °C, 45 s), with a final extension (72 °C, 10 min). PCR products were purified using AMPure XP beads (Beckman Coulter Genomics, Danvers, MA, USA) and quantified using a Qubit fluorometer (Invitrogen). PCR products were evaluated using the Illumina Library Quantification Kit (Kapa Biosciences, Woburn, MA, USA). Sequencing was performed by LC-Bio Technologies Co., Ltd., using an Illumina NovaSeq 6000 platform (Illumina, CA, United States; Yang et al., 2024).

### 2.6 Bioinformatic analysis of sequencing data

Bioinformatics analyses were performed by LC-Bio Technologies Co., Ltd. (Hangzhou, China). For the bacterial 16S rRNA gene sequences, 80,683–87,536 raw reads were obtained per sample, whereas 81,196–87,718 raw reads were obtained per sample for fungal internal transcribed spacer (ITS) sequences. Sequencing primers were removed from the demultiplexed raw sequences using Cutadapt (version 1.9). Paired-end reads were merged using the FLASH (version 1.2.8) or PEAR (version 0.9.6) software. The low-quality reads (quality scores <20), short reads (<100 bp), and reads containing >5% “N” records were trimmed using the sliding-window algorithm method in fqtrim (version 0.94). Quality filtering was performed to obtain high-quality clean tags according to fqtrim. For the bacterial 16S rRNA gene sequences, 65,897–75,936 reads per sample were retained after quality filtering, whereas 77,201–83,775 reads per sample were retained for fungal ITS sequences. Potential chimeric sequences were identified and removed using VSEARCH (version 2.3.4) and the reference-based UCHIME method (−-uchime_ref). The RDP Gold reference database was used for bacterial 16S rRNA gene sequences, and the ITS2-specific reference database was used for fungal ITS sequences. The resulting non-chimeric sequences were subsequently denoised using the dada2 denoise-single workflow in QIIME 2 (version 2024.10; Callahan et al., 2016; Bolyen et al., 2019). For bacterial 16S rRNA gene sequences, the parameters were set to the--p-trunc-len 400 --p-max-ee 2.0 --p-chimera-method none, whereas for fungal ITS sequences, the --p-trunc-len 250 --p-max-ee 2.0 --p-chimera-method none were used. Amplicon sequence variant (ASV) representative sequences and corresponding abundance tables were generated, and singleton ASVs, defined as ASVs with a total abundance of only one read across all samples, were removed. For bacterial and fungal sequencing data, six samples were retained for analysis, with sequencing depths ranging from 55,221 to 66,708 reads per sample for bacterial 16S rRNA gene sequencing and from 32,241 to 48,191 reads per sample for fungal ITS sequencing. Bacterial taxonomic assignment was conducted using the SILVA (version 138; Quast et al., 2013) and NT-16S databases, whereas fungal taxonomic classification was performed using the RDP (version 2.12) and UNITE (release 2024.04.04) databases (Lan et al., 2012). Subsequently, a phylogenetic tree was constructed to characterize the evolutionary relationships among ASVs. The resulting ASV feature table, representative sequences, taxonomic annotation table, and phylogenetic tree were used for downstream analysis.

### 2.7 Statistical analysis

The yield, bioactive compound content, and soil chemical property data were subjected to significance testing using RStudio (R version 4.2.3) and plotted using OriginPro (version 2025b). For each soil chemical property and bioactive compound, a linear mixed-effects model was fitted with the cropping system and developmental stage as fixed effects and plot as a random effect. Type III analysis of variance (ANOVA) was used to evaluate the significance of treatment, stage, and their interactions. The estimated marginal means were used for pairwise comparisons between treatments within each developmental stage, with *p* values adjusted using the Holm method (*p* < 0.05). For the microbial sequencing data, the rarefaction depth was set to the lowest sequencing depth among all samples to ensure a consistent sequencing depth across samples while retaining as much sample information as possible. The ASV abundance tables for bacteria and fungi were separately rarefied using the phyloseq package (version 1.52.0; Wen et al., 2023). The rarefaction depths were 55,221 reads per sample for bacteria and 32,241 reads per sample for fungi, retaining 93.06 and 75.03% of the total number of reads before rarefaction, respectively. The rarefied ASV abundance tables were subsequently used for Venn diagrams, chord diagrams, α-diversity, beta-diversity, functional prediction, and correlation analyses. Subsequently, alpha diversity indices were calculated using the microbiome package (version 1.30.0; Koh et al., 2021), and visualizations were produced using ggplot2 (version 4.0.0). Beta diversity was assessed using Bray–Curtis dissimilarities. Principal coordinate analysis (PCoA) was used to visualize the differences in the microbial community composition between treatments, and permutational multivariate analysis of variance was performed to test for treatment effects. The homogeneity of multivariate dispersion was assessed using the betadisper function and a permutation test. Differentially abundant microbial taxa between the continuous cropping and rotation treatments were identified using the Linear Discriminant Analysis Effect Size (LEfSe) method implemented in OmicStudio.^1^ The original ASV abundance table was used as the input, and total sum scaling (TSS) normalization was applied to convert ASV abundances to relative abundances prior to LEfSe analysis. LEfSe was performed by sequentially applying the Kruskal–Wallis rank-sum test (*p* < 0.05), Wilcoxon rank-sum test (*p* < 0.05), and linear discriminant analysis (LDA) to identify taxa showing significant differences between treatments and to estimate their effect sizes. Taxa with LDA scores > 3.5 were considered potential discriminative biomarkers (*p* < 0.05; Khleborodova et al., 2024). Functional profiles of bacterial and fungal communities were predicted using the FAPROTAX database (Louca et al., 2016) and FUNGuild (Nguyen et al., 2016), respectively, using the Wekemo Bioincloud platform.^2^ The resulting predicted functional profiles were normalized using the TSS method. Normality was assessed using the Shapiro–Wilk test, and homogeneity of variances was evaluated using Levene’s test. For each predicted functional classification, an independent-samples t-test was performed when both groups met the normality criterion (*p* > 0.05) and homogeneity of variance criterion (*p* > 0.05); alternatively, the Wilcoxon rank-sum test was used. *p*-values from all predicted functional classification comparisons were adjusted using the Benjamini–Hochberg method. Comparisons of the relative abundances of microbial taxa at the phylum level were performed using the same statistical procedures. Microbial chord diagrams were generated using the Circlize package (version 0.4.12; Su, 2025). Spearman’s rank correlation analysis was performed using the corrplot package (version 0.89) to assess the correlations between soil chemical properties and biomarkers identified by LEfSe analysis and the yield and quality of *A. chinensis*. *p*-values were adjusted using the Benjamini–Hochberg method.

## 3 Results

### 3.1 Effects of continuous cropping on the yield of *Atractylodes chinensis*

The two cropping systems exhibited significant differences in *A. chinensis* yield (Figure 1). Continuous cropping significantly reduced yield by 12.7% compared to that of crop rotation, decreasing from 7359.58 to 6422.08 kg/hm^2^.

**Figure 1:**
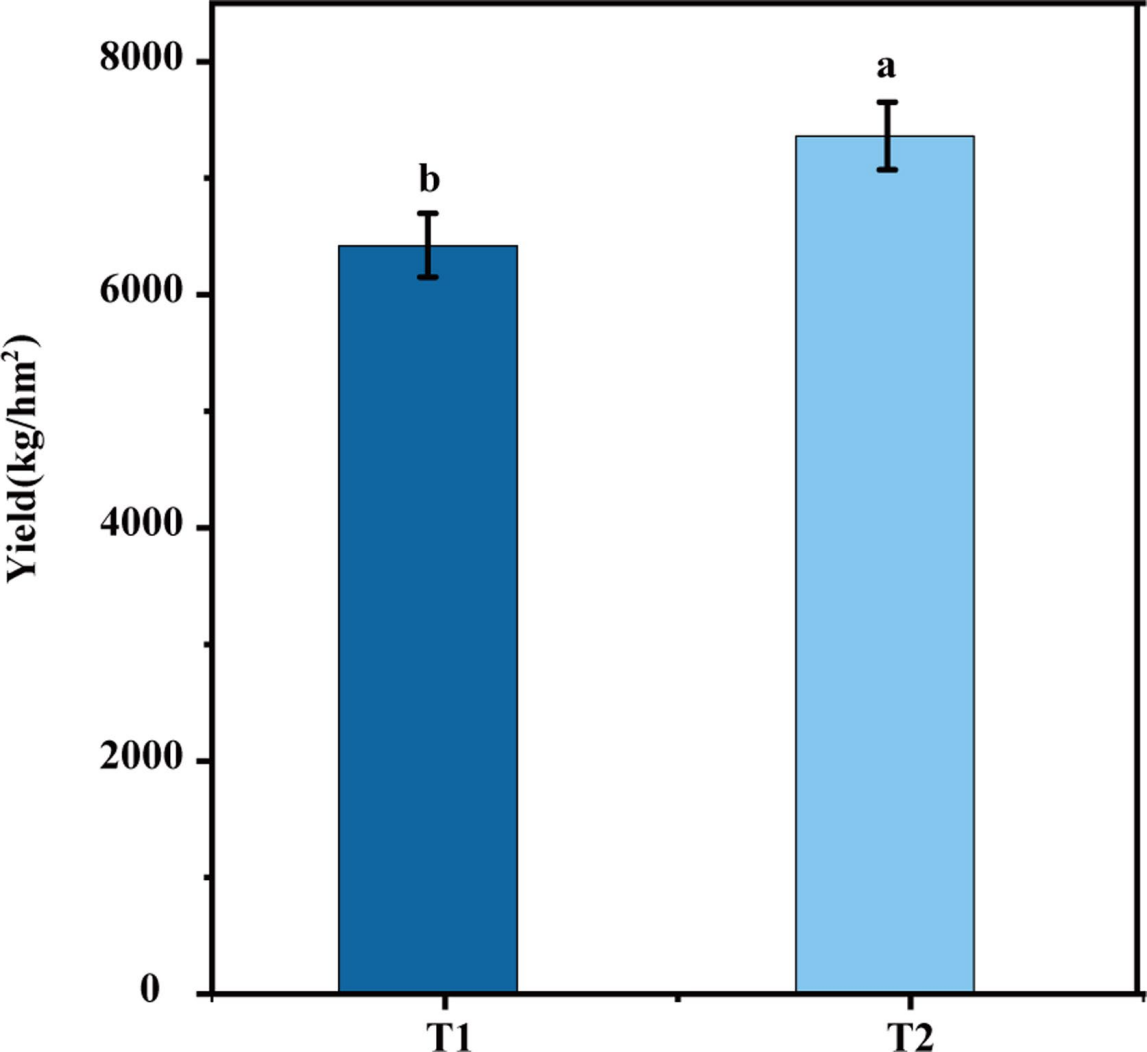
Effects of continuous cropping on the yield of *Atractylodes chinensis*. T1, continuous cropping; T2, crop rotation. Bars represent mean ± standard deviation (SD; *n* = 3). Different letters indicate significant differences between treatments based on an independent-samples *t*-test (*p* < 0.05).

### 3.2 Effects of continuous cropping on bioactive compound accumulation in *Atractylodes chinensis*

Linear mixed-effects models were fitted separately for the three major bioactive compounds (β-eudesmol, atractylodin, and atractylon), with cropping system and growth stage as fixed effects and plot as a random effect. The growth stage had highly significant effects on the content of all three compounds (*p* < 0.001), indicating that their content varied dynamically throughout plant development. Across the entire growth period, all three compounds exhibited a similar pattern; their content first increased, then decreased, and subsequently increased again, with the highest levels consistently observed at the flowering stage (Figure 2). In addition, the cropping system × growth stage interaction significantly affected β-eudesmol content, whereas the main effect of cropping system was not significant. Further pairwise comparisons between cropping systems within each growth stage showed that, compared with crop rotation, continuous cropping significantly increased β-eudesmol content at the leaf-expansion stage and significantly decreased it at the flowering, fruiting, and harvest stages, with reductions of 8.33%, 16.84%, and 21.25%, respectively (Figure 2A). The cropping system (*p* = 0.029), growth stage (*p* < 0.001), and their interaction (*p* < 0.001) significantly affected the atractylodin content. Atractylodin content was significantly higher under continuous cropping than under crop rotation at the leaf expansion and flowering stages; however, this pattern was reversed from the fruiting stage onward. The atractylodin content under continuous cropping was significantly lower by 24.4 and 10.39% at the fruiting and harvest stages, respectively, than under crop rotation (Figure 2B). Atractylon content was significantly affected by cropping system (*p* < 0.001) and growth stage (*p* < 0.05); however, their interaction was not significant. Overall, the atractylon content was lower under continuous cropping than under rotation (Figure 2C).

**Figure 2:**
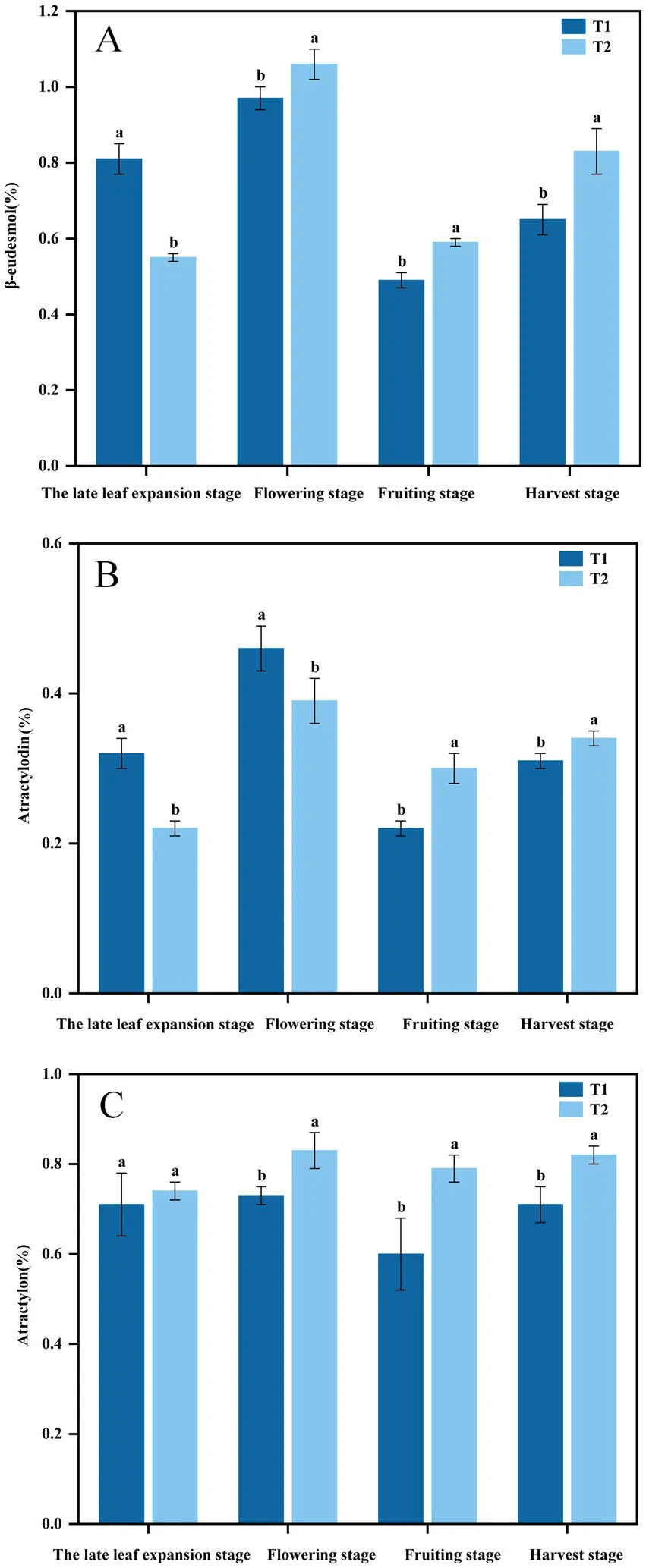
Effects of continuous cropping on the bioactive compound contents in *A. chinensis*. **(A)** β-eudesmol content; **(B)** Atractylodin content; **(C)** Atractylon content. T1, continuous cropping; T2, crop rotation. Bars represent mean ± SD (*n* = 3). For each parameter, different lowercase letters within each developmental stage indicate significant differences between treatments, based on the estimated marginal means from a linear mixed-effects model, with *p*-values adjusted using the Holm method for multiple comparisons (*p* < 0.05).

### 3.3 Effects of continuous cropping on rhizosphere soil chemical properties in *Atractylodes chinensis*

No significant differences were observed in the soil chemical properties between the T1 and T2 plots before the initiation of the experiment (*p* > 0.05; Supplementary Table S1). Following continuous cropping and rotation treatments, rhizosphere soils were collected at different growth stages of *A. chinensis* to determine the soil chemical properties. Linear mixed-effects models were fitted separately for each soil variable, with the cropping system and growth stage as fixed effects and plot as a random effect. Type III analysis of variance was used to test the significance of the main effects of cropping system, growth stage, and their interaction, followed by *post hoc* pairwise comparisons between treatments within each growth stage based on estimated marginal means, with *p*-values adjusted using the Holm method for multiple comparisons. The soil in the experimental area was slightly alkaline (Table 1). The mixed-effects model showed that the cropping system (*p* < 0.001) and growth stage (*p* < 0.001) had highly significant effects on soil pH, whereas their interaction was not significant. Overall, soil pH was significantly higher under continuous cropping than under rotation and gradually increased across the growth stages. Soil EC was significantly affected by cropping system (*p* < 0.001), growth stage (*p* < 0.001), and their interaction (*p =* 0.020) and increased across growth stages. Further pairwise comparisons between cropping systems within each growth stage showed that the EC values under continuous cropping were significantly higher than those under crop rotation at the flowering, fruiting, and harvest stages (*p* < 0.05), with increases of 8.64%, 22.84%, and 15.49%, respectively, compared to those of the crop rotation treatment. According to the criteria of the Second National Soil Survey of China, AN and AK contents in the experimental area were relatively low. AN was significantly affected by cropping system (*p* < 0.001), growth stage (*p* < 0.001), and their interaction (*p* = 0.040) and gradually decreased across growth stages. Post-hoc comparisons showed that AN content was consistently lower under continuous cropping than under rotation at all growth stages, with reductions of 10.08%, 17.09%, 19.40%, and 9.00%, respectively. AK was significantly affected by cropping system, growth stage, and their interactions (*p* < 0.05, *p* < 0.05, and *p* = 0.022, respectively). The AK content was significantly higher under continuous cropping than under rotation during the fruiting and harvest stages, whereas no significant difference was observed during the leaf expansion and flowering stages. AP remained at a relatively high level. The main effect of the cropping system was not significant (*p* = 0.264), whereas the effects of growth stage (*p* < 0.001) and the cropping system × growth stage interaction (*p* < 0.001) were highly significant. The OM content was significantly affected by cropping system (*p* = 0.005) and the interaction (*p* = 0.001). At the fruiting (*p* < 0.001) and harvest stages (*p* = 0.008), the OM content was significantly lower in the continuous cropping treatment than in the rotation treatment.

**Table 1:** Effects of continuous cropping on rhizosphere soil chemical properties of *Atractylodes chinensis*.

Soil variable	Cropping systems	The late leaf expansion stage	The flowering stage	The fruiting stage	The harvest stage
pH	T1	8.33 ± 0.04a	8.40 ± 0.07a	8.39 ± 0.02a	8.51 ± 0.04a
	T2	8.22 ± 0.06b	8.27 ± 0.04b	8.35 ± 0.01a	8.41 ± 0.02b
EC (μS/cm)	T1	65.67 ± 2.52a	67.00 ± 2.65a	80.67 ± 3.06a	82.00 ± 1.00a
	T2	61.33 ± 4.51a	61.67 ± 0.58b	65.67 ± 4.16b	71.00 ± 2.65b
AN (mg/kg)	T1	56.23 ± 2.04b	46.43 ± 2.38b	44.57 ± 1.36b	40.13 ± 2.07b
	T2	62.53 ± 2.84a	56.00 ± 2.04a	55.30 ± 1.80a	44.10 ± 1.10a
AP (mg/kg)	T1	35.66 ± 3.98b	43.43 ± 0.65a	35.50 ± 0.34a	30.32 ± 1.96a
	T2	45.11 ± 1.26a	39.66 ± 2.21b	33.66 ± 1.54a	30.69 ± 0.65a
AK (mg/kg)	T1	47.83 ± 1.38a	46.24 ± 6.91a	58.21 ± 2.77a	52.62 ± 3.66a
	T2	42.25 ± 2.39a	44.64 ± 2.69a	43.38 ± 1.47b	45.31 ± 3.59b
OM (g/kg)	T1	9.80 ± 1.00a	8.68 ± 1.06a	4.85 ± 1.29b	8.09 ± 0.72b
	T2	10.66 ± 0.87a	10.28 ± 1.74a	12.56 ± 1.84a	11.23 ± 1.19a

T1, continuous cropping; T2, crop rotation. EC, electrical conductivity; AN, alkaline hydrolysis nitrogen; AP, available phosphorus; AK, available potassium; OM, organic matter. Values are means ± SD (*n* = 3). For each parameter, different lowercase letters within each developmental stage indicate significant differences between treatments, based on the estimated marginal means from a linear mixed-effects model, with *p*-values adjusted using the Holm method for multiple comparisons (*p* < 0.05).

### 3.4 Effects of continuous cropping on rhizosphere microbial community composition in *Atractylodes chinensis*

High-throughput sequencing was performed to characterize the rhizosphere microbial communities in different cropping systems (Figure 3). A total of 7,207 bacterial ASVs were identified in the continuous cropping treatment and 6,668 ASVs in the crop rotation treatment. Specifically, 4,444 and 3,905 ASVs were unique to continuous cropping and crop rotation, respectively, whereas only 2,763 ASVs were shared between the two treatments (Figure 3A). At the phylum level, Actinobacteria, Chloroflexi, Planctomycetota, Proteobacteria, and Acidobacteria were the dominant bacterial taxa in both treatments, with no significant differences in their relative abundances between the treatments (Figure 3C). For the fungal communities, 757 and 895 ASVs were identified under continuous cropping and crop rotation, respectively. Of these, 419 and 557 ASVs were unique to each treatment, whereas 338 ASVs were shared between the two treatments (Figure 3B). Community composition analysis revealed that Ascomycota, Basidiomycota, and Zygomycota were the dominant fungal phyla. Compared to crop rotation, continuous cropping significantly reduced the relative abundance of Basidiomycota (T1: 6.85%; T2: 17.16%; Figure 3D). In addition, PCoA based on Bray–Curtis dissimilarities showed no significant differences in the overall bacterial or fungal community composition between the two treatments (Supplementary Figures S1A,B).

**Figure 3:**
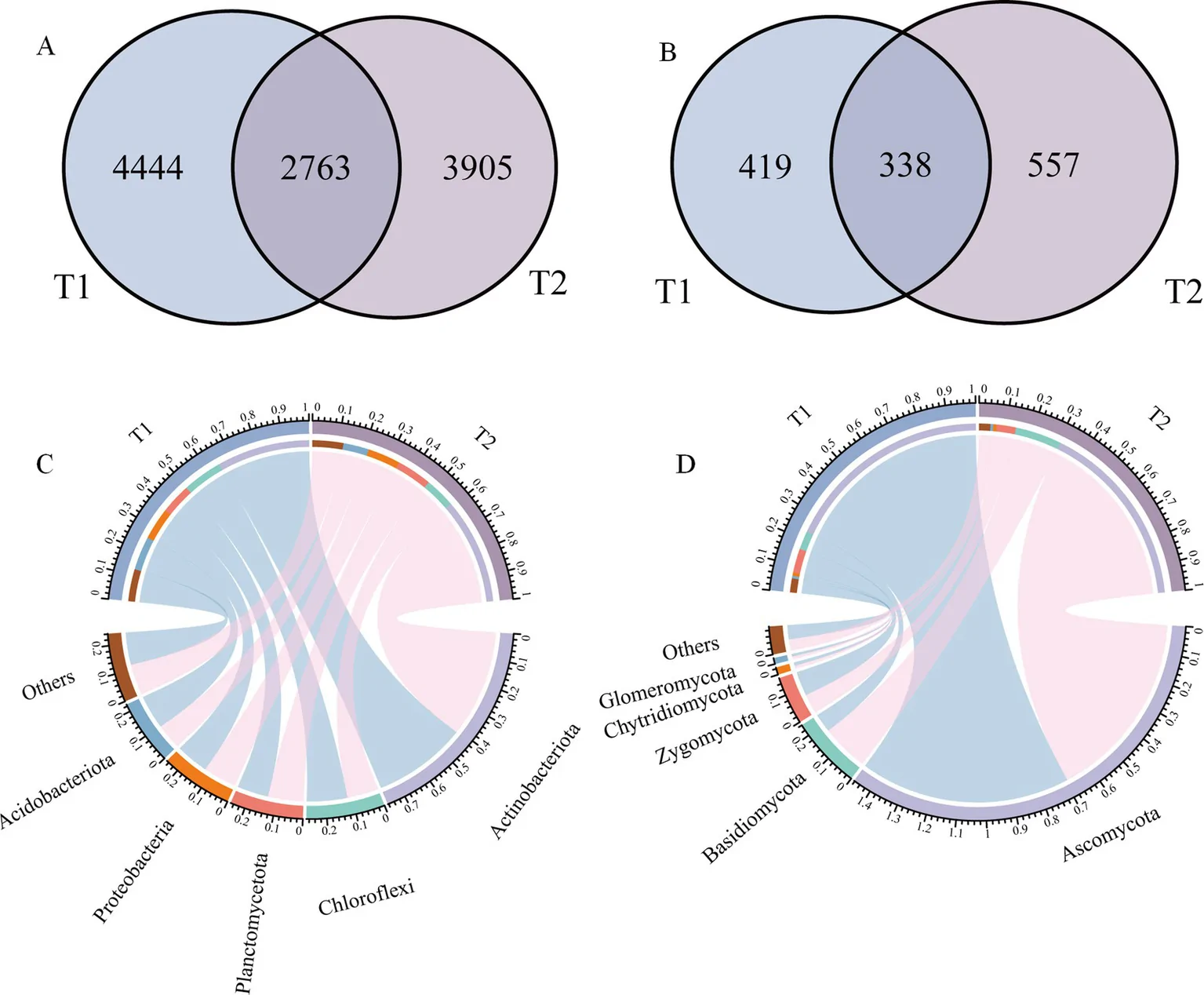
Rhizosphere microbial community composition of *A. chinensis* in continuous cropping and crop rotation systems. **(A)** Shared and unique bacterial amplicon sequence variant (ASV) between treatments. **(B)** Shared and unique fungal ASVs between treatments. **(C)** Relative abundances of dominant bacterial phyla visualized using chord diagrams. **(D)** Relative abundances of dominant fungal phyla visualized using chord diagrams. T1, continuous cropping; T2, crop rotation.

### 3.5 Effects of continuous cropping on rhizosphere microbial alpha diversity in *Atractylodes chinensis*

Differences in rhizosphere microbial alpha diversity between continuous cropping and crop rotation were evaluated (Figure 4). Compared with crop rotation, continuous cropping significantly increased the Shannon diversity index and Pielou’s evenness of bacterial communities, whereas no significant difference was observed in the Chao1 index, indicating that continuous cropping altered bacterial community evenness without significantly affecting species richness (Figures 4A–C). In contrast, continuous cropping significantly decreased the Chao1 index of the fungal communities, whereas no significant differences were observed in the Shannon diversity index or Pielou’s evenness (Figures 4D–F). These results indicate that continuous cropping reduced fungal species richness; however, it had a limited effect on fungal community evenness.

**Figure 4:**
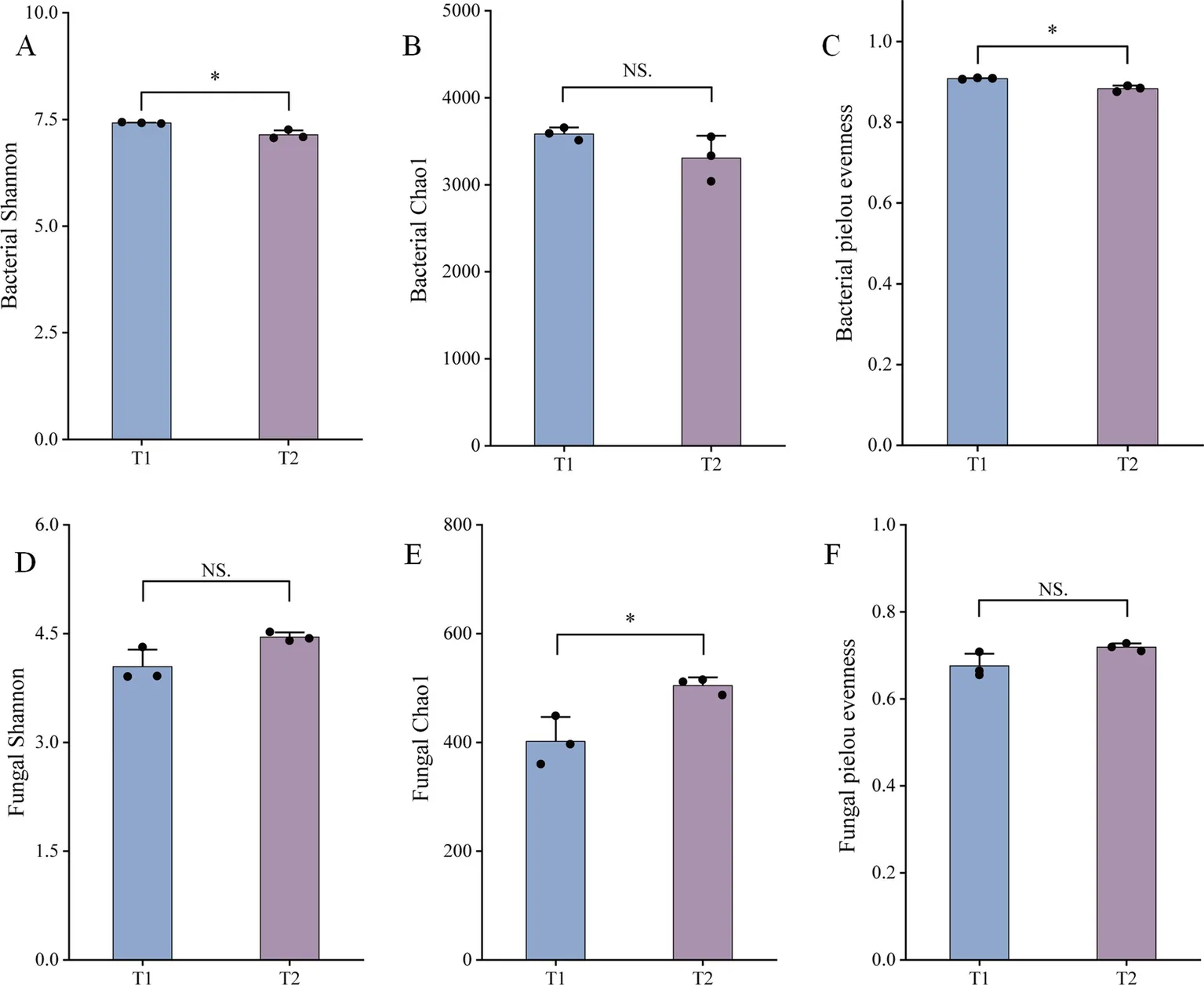
Effects of continuous cropping on the alpha diversity of rhizosphere microbial communities in *A. chinensis*. **(A)** Shannon index of bacterial communities, **(B)** Chao1 index of bacterial communities, **(C)** Pielou’s evenness index of bacterial communities, **(D)** Shannon index of fungal communities, **(E)** Chao1 index of fungal communities, and **(F)** Pielou’s evenness index of fungal communities. The asterisk above the bars indicates a significant difference between two independent sample *t*-tests (*p* < 0.05).

### 3.6 Differential microbial taxa in the rhizosphere of *Atractylodes chinensis*

The LEfSe analysis revealed distinct discriminatory taxa between the two cropping systems at multiple taxonomic levels (Figure 5). At the family level, distinct taxa were enriched in continuous cropping and crop rotation. Specifically, bacterial taxa such as Vicinamibacteraceae and fungal taxa including Dothidotthiaceae and Nectriaceae were enriched under continuous cropping, whereas bacterial taxa such as Nocardioidaceae and Micrococcaceae and fungal taxa including Pseudombrophilaceae and Pyronemataceae were enriched under crop rotation. Several key microbial taxa that are closely associated with cropping systems were identified at the genus level. Continuous cropping significantly enriched fungal genera, such as *Fusarium* and *Volutella*; however, it reduced the abundance of bacterial genera, including *Nocardioides* and *Arthrobacter*, and fungal genera, such as *Aleuria* and *Heydenia*.

**Figure 5:**
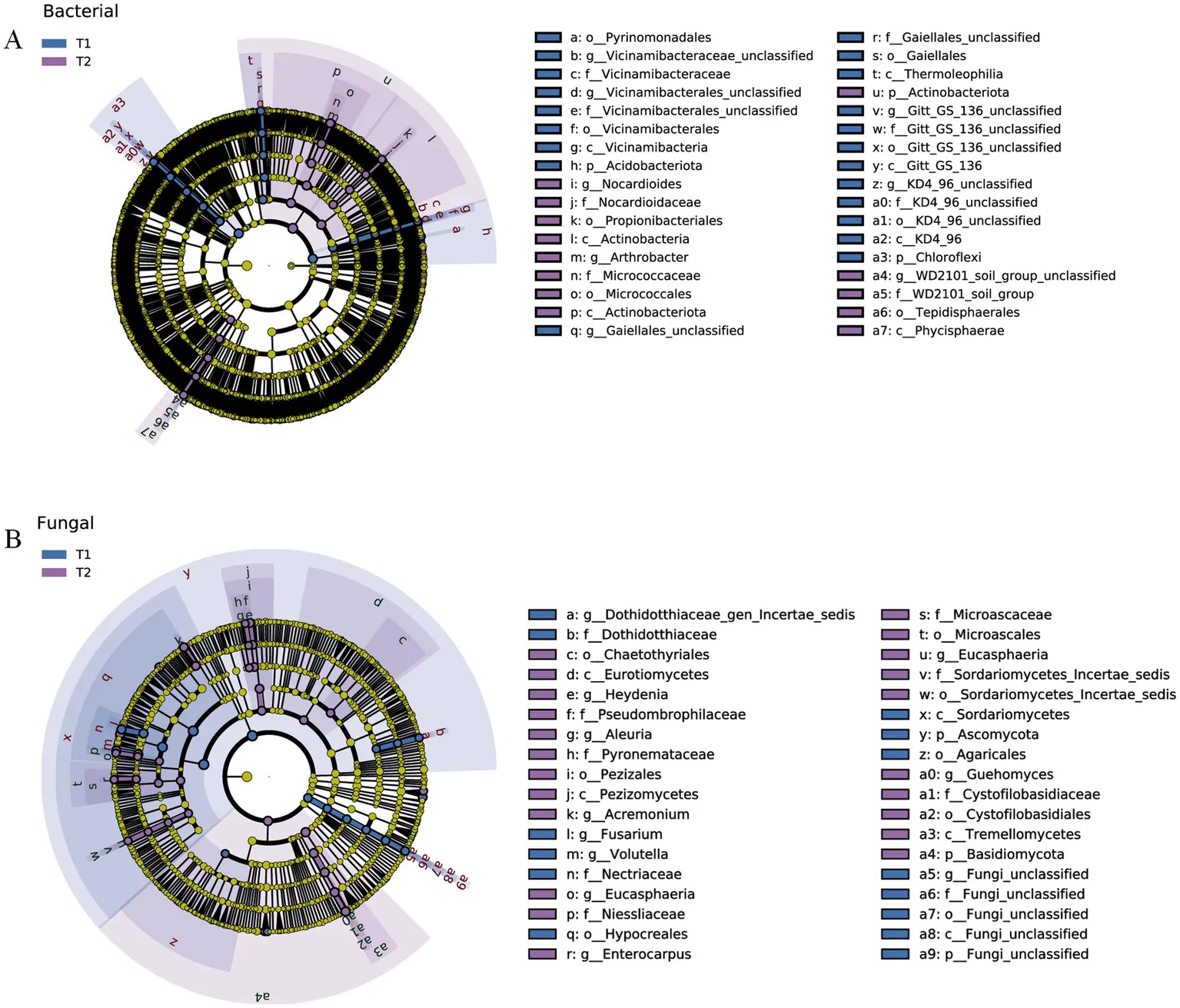
Linear discriminant analysis effect size (LEfSe) analysis of the rhizosphere microbial communities in *A. chinensis* under different cropping systems. **(A)** Bacterial and **(B)** fungal communities. Cladogram showing significant enrichment of taxonomic groups in each treatment. Only taxa with linear discriminant analysis (LDA) scores > 3.5 and *p* < 0.05 are shown.

### 3.7 Functional prediction of rhizosphere microbial communities

Functional predictions based on the FAPROTAX database revealed significant differences in the predicted bacterial functional profiles between the two cropping systems (Figure 6A). Compared to crop rotation, continuous cropping significantly decreased the relative abundance of several predicted functional classifications associated with the carbon cycle, including ligninolysis, methanol oxidation, and methylotrophy. In addition, the relative abundances of several predicted functional classifications associated with the nitrogen cycle, including nitrate respiration, nitrite respiration, nitrate reduction, and nitrogen respiration, were significantly reduced under continuous cropping. The FUNGuild database was used to predict the functional profiles of fungal communities (Figure 6B). Three major trophic modes were identified, including saprotrophs, symbiotrophs, and pathotrophs; however, no significant differences were observed among them.

**Figure 6:**
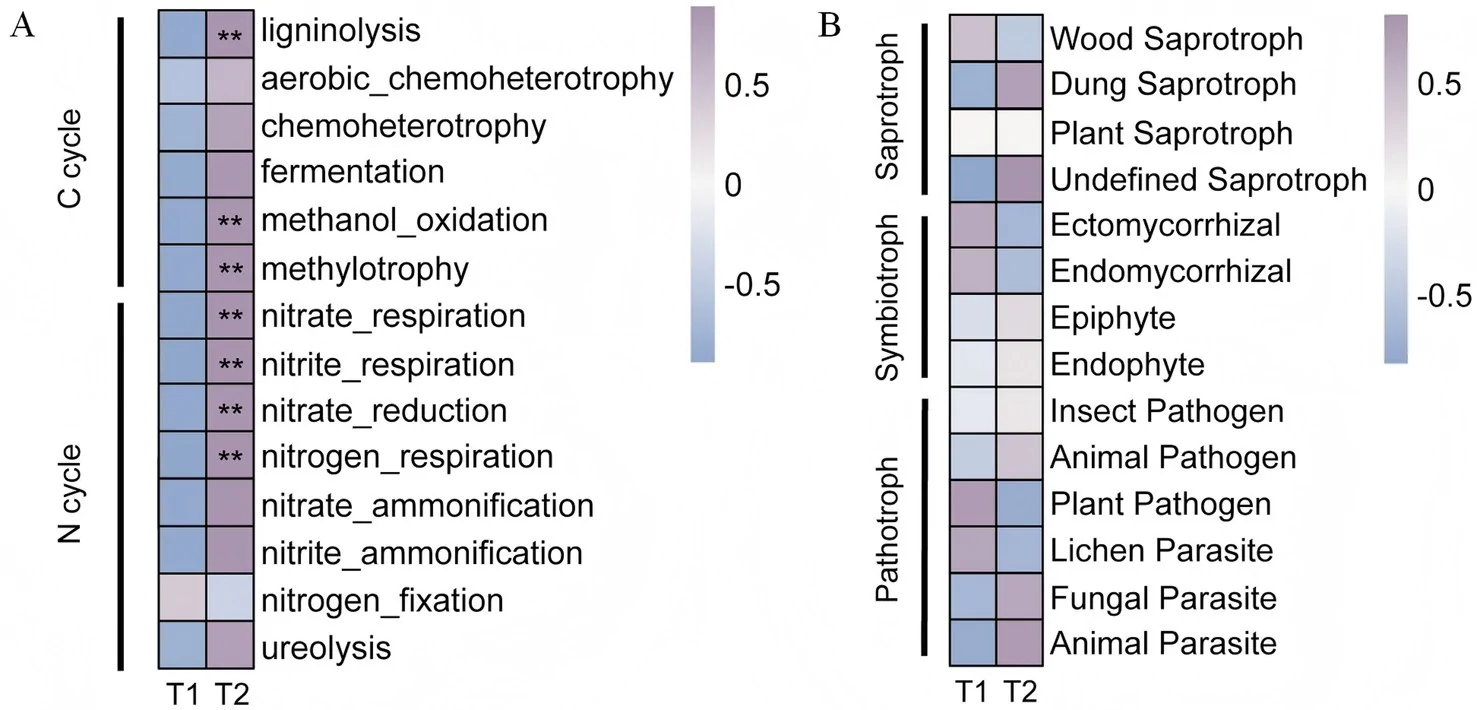
Functional prediction of the rhizosphere microbial communities in *A. chinensis* under different cropping systems. **(A)** Predicted bacterial functional profiles based on FAPROTAX analysis. **(B)** Predicted fungal functional profiles based on FUNGuild. Asterisks indicate significant differences between treatments (*p < 0.05; ** *p* < 0.01).

### 3.8 Associations among soil chemical properties, microbial biomarkers, yield, and quality traits

To investigate the statistical associations among rhizosphere soil chemical properties, LEfSe-identified microbial biomarkers, and the yield and quality traits of *A. chinensis*, Spearman’s rank correlation analysis was performed for six soil chemical properties (pH, EC, AN, AP, AK, and OM), 10 LEfSe-identified differential genera with distinct taxonomic assignments (*Nocardioides*, *Arthrobacter*, *Heydenia*, *Aleuria*, *Acremonium*, *Fusarium*, *Volutella*, *Eucasphaeria*, *Enterocarpus*, and *Guehomyces*), yield, and three bioactive compounds (atractylodin, atractylon, and β-eudesmol; Figure 7). Among the soil chemical properties, pH was significantly negatively correlated with AN (*r* = −1, *p* < 0.001), whereas EC was significantly negatively correlated with OM (*r* = −1, *p* < 0.001). Several microbial taxa were significantly associated with soil chemical properties. *Heydenia* was significantly negatively correlated with EC (*r* = −1, *p* < 0.001) and positively correlated with OM (*r* = 1, *p* < 0.001). Similarly, *Enterocarpus* was significantly negatively correlated with EC (*r* = −0.94, *p* < 0.05) and positively correlated with OM (*r* = 0.94, *p* < 0.05). *Volutella* was significantly and positively correlated with AK (*r* = 0.94, *p* < 0.05). Among the microbial taxa, *Nocardioides* was significantly positively correlated with *Arthrobacter* (*r* = 1, *p* < 0.001), whereas *Nocardioides*, *Arthrobacter*, and *Guehomyces* were significantly negatively correlated with Volutella (*r* = −0.94, *p* < 0.05). Additionally, *Enterocarpus* was significantly positively correlated with *Heydenia* (*r* = 0.94, *p* < 0.05), whereas *Fusarium* was significantly negatively correlated with *Aleuria* (*r* = −0.94, *p* < 0.05). Regarding plant performance, yield was significantly negatively correlated with EC (*r* = −1, *p* < 0.001) and positively correlated with OM (*r* = 1, *p* < 0.001) and the relative abundances of *Heydenia* (*r* = 1, *p* < 0.001) and *Enterocarpus* (*r* = 0.94, *p* < 0.05). For quality traits, atractylon content was significantly positively correlated with the relative abundances of *Aleuria* and *Acremonium* (both *r* = 0.94, *p* < 0.05) and significantly negatively correlated with the relative abundance of *Volutella* (*r* = −0.94, *p* < 0.05). Spearman’s correlation analysis indicated that several soil chemical properties and specific microbial taxa were significantly associated with the yield and quality traits of *A. chinensis*.

**Figure 7:**
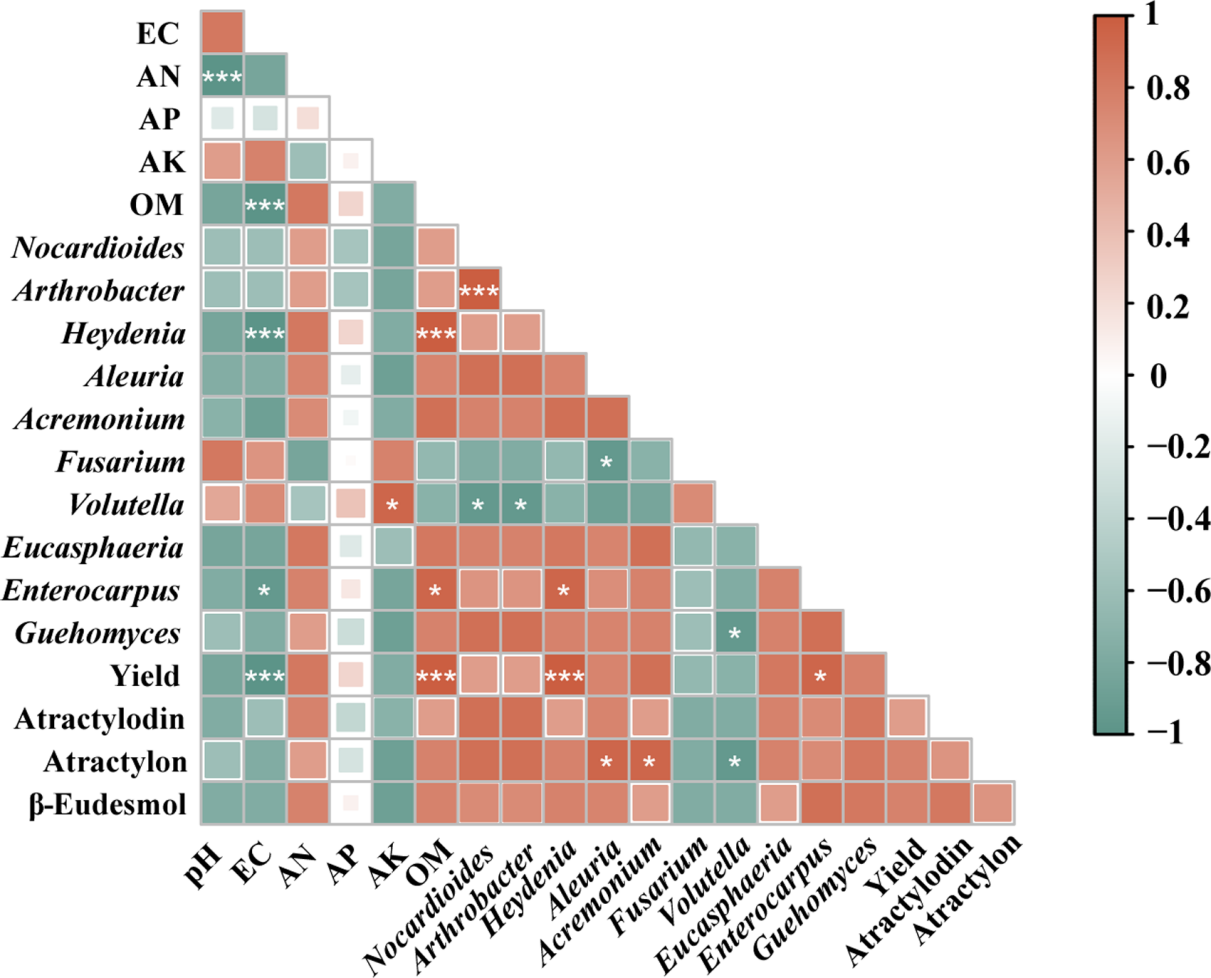
Associations among soil chemical properties, microbial biomarkers, yield, and quality traits of *A. chinensis* based on Spearman’s correlation analysis.

## 4 Discussion

Continuous cultivation of the same or closely related medicinal plants on the same land frequently results in a substantial decline in yield and quality, a phenomenon commonly referred to as a CCO (Chen et al., 2022). However, the specific effects of continuous cropping remain poorly understood, as they vary considerably among plant species and ecological environments. Continuous cropping induces a series of adverse effects, including accumulation of autotoxic compounds, deterioration of soil physicochemical properties, and disruption of soil microbial communities (Pervaiz et al., 2020). These changes impair plant growth and reduce yield and quality (An et al., 2022). In the present study, continuous cropping significantly reduced the yield of *A. chinensis*, which is consistent with previous findings for other medicinal plants such as *Rehmannia glutinosa* (Gan et al., 2025) and *Curcuma kwangsiensis* (Huang et al., 2025). Additionally, the levels of the three major bioactive compounds, atractylodin, atractylon, and β-eudesmol, decreased by 8.33–21.25% under continuous cropping. These findings indicate that continuous cropping of *A. chinensis* in the Horqin Sandy Land may induce CCOs, as evidenced by substantial reductions in yield and medicinal quality.

Long-term continuous cropping results in the deterioration of soil chemical properties (Liu et al., 2021). In the present study, continuous cropping significantly increased soil EC and pH, indicating elevated levels of soluble salt ions and increased soil alkalinity. This may be attributed to the accumulation of root exudates during long-term continuous cropping (Shen et al., 2025). Continuous cropping altered soil nutrient dynamics. Prolonged continuous cultivation depletes essential nutrients required by crops, while promoting the accumulation of nutrients that plants cannot efficiently assimilate, thereby resulting in nutrient imbalance (Zhao et al., 2024). Consistent with these findings, continuous cropping significantly reduced soil AN and OM contents in the rhizosphere of *A. chinensis*, similar to observations in *Gastrodia elata* (Feng et al., 2026). AK levels increased during certain developmental stages, which is consistent with findings reported for *A. macrocephala* (Chen et al., 2014). This increase may be related to changes in the microbial community that promote mineral K release under continuous cropping stress (Sun et al., 2023). An alternative explanation is that growth inhibition under continuous cropping may reduce plant K demand and potentially contribute to the transient accumulation of K in rhizosphere soil. These findings highlight the complexity of soil nutrient dynamics and indicate that nutrient responses to continuous cropping vary across environmental contexts.

Soil microbial diversity is critical in maintaining agricultural ecosystem stability. In the present study, continuous cropping significantly reduced fungal richness in the rhizosphere of *A. chinensis*, which is consistent with observations in *Coptis chinensis* Franch., *Beta vulgaris* L., and *Apium graveolens* L. (Song et al., 2018; Xu et al., 2025; Lu et al., 2023). The accumulation of root exudates and the decomposition of plant residues under continuous cropping may selectively enrich pathogenic fungi, thereby occupying ecological niches and reducing the overall fungal richness. In contrast, bacterial richness remained relatively stable, whereas bacterial evenness increased, suggesting that previously dominant taxa may have been suppressed, resulting in a more homogeneous bacterial community structure.

PCoA based on Bray–Curtis dissimilarities showed no significant differences in the overall bacterial or fungal community composition between the two treatments. However, a substantial number of treatment-specific ASVs were identified, suggesting that, although the overall community composition did not differ significantly, certain taxa may vary in relative abundance between the two cropping systems. The LEfSe analysis further supported this observation. Compared to crop rotation, continuous cropping significantly enriched *Nectriaceae* and its representative genera, *Fusarium* and *Volutella*. Certain strains within these genera are major pathogenic agents associated with *A. chinensis* root rot (Luo et al., 2023; Xie et al., 2024). Therefore, the enrichment of *Fusarium* and *Volutella* observed in the present study may indicate an increased relative abundance of potentially pathogenic taxa, which may be associated with increased disease risk under continuous cropping. Additionally, continuous cropping significantly reduced the relative abundance of *Nocardioides* and *Arthrobacter*. Certain strains within the genus *Nocardioides* exhibit functions such as phosphorus solubilization, siderophore production, nitrogen fixation, indole-3-acetic acid (IAA) production, and degradation of deoxynivalenol produced by *Fusarium* species (Saikia et al., 2022; Meena et al., 2020; Xiao et al., 2021; Zhang et al., 2021). Similarly, certain *Arthrobacter* strains exhibit plant growth-promoting potential, including the production of proteases, amylases, IAA, and siderophores, as well as lignin and cellulose degradation capabilities (Wang et al., 2026; Liao et al., 2026; Giannelli et al., 2022). The reduction in these potentially beneficial microbial taxa may be associated with the weakened soil nutrient cycling and disruption of soil microbial balance. The functional prediction analysis produced similar results. Continuous cropping significantly reduced the relative abundances of several predicted microbial functional profiles related to carbon and nitrogen cycling, indicating that the predicted functional potential for microbially mediated carbon metabolism and nitrogen transformation may be reduced. This pattern was consistent with the reduced relative abundances of the potentially beneficial taxa *Nocardioides* and *Arthrobacter*, suggesting that continuous cropping may impair the microbial-mediated functional potential of carbon and nitrogen cycling, which potentially affects soil nutrient availability and ecosystem stability.

Spearman’s correlation analysis was used to evaluate the associations among soil chemical properties, microbial biomarkers, and yield and quality indicators of *A. chinensis*. Soil chemical properties directly influence plant growth and development (Fierer, 2017; Tedersoo et al., 2014). In the present study, soil OM content was significantly and positively correlated with yield. In contrast, EC was significantly and negatively correlated with both yield and OM content, indicating that OM and EC are important environmental factors related to the growth performance of *A. chinensis*. OM is a key parameter of soil fertility (Zech et al., 1997) that maintains ecological functions and crop productivity (Sarker et al., 2018), whereas EC indicates the total soluble salt ion concentration in the soil. In continuous cropping studies of *A. macrocephala*, elevated soil EC has been confirmed as an important indicator of soil degradation; high EC modifies rhizosphere fungal and bacterial community structures, aggravates root growth impairment, and is closely associated with root decline and yield reduction in *Atractylodes* species (Zhu et al., 2020). In addition to soil properties, several biomarkers were significantly associated with yield, which was positively correlated with the relative abundances of *Heydenia* and *Enterocarpus*. Certain *Heydenia* species antagonize plant pathogens and exhibit ACC deaminase activity (Yang et al., 2020), indicating potentially beneficial plant-associated functions that may contribute to yield. *Enterocarpus* was predicted to have a saprotrophic trophic mode based on functional predictions in the present study (Nguyen et al., 2016), and its relative abundance was positively correlated with the OM content. These findings suggest that *Enterocarpus* may be involved in OM decomposition, which may contribute to nutrient availability in the rhizosphere and indirectly support yield formation. However, because FUNGuild assigns putative ecological functions based mainly on taxonomic information rather than on direct measurements of microbial activity, the specific contribution of *Enterocarpus* to OM decomposition, nutrient availability, and plant growth requires experimental validation. Furthermore, certain microbial taxa were significantly associated with the bioactive compound contents in *A. chinensis*, which is consistent with previous studies on *A. chinensis* (Bai et al., 2025). In the present study, atractylon content was significantly positively correlated with *Acremonium* and *Aleuria* and significantly negatively correlated with *Volutella*. *Acremonium* species exhibit diverse ecological functions, and certain strains promote plant secondary metabolism and growth or directly produce secondary metabolites (Han et al., 2020; Qin et al., 2024), which is consistent with the positive *Acremonium*–atractylon correlation observed in the present study, suggesting a potential association between this fungal group and secondary metabolite accumulation in *A. chinensis*. Studies directly linking *Aleuria* to the growth or metabolism of medicinal plants remain limited; however, isotopic evidence suggests that certain strains exhibit a mycorrhizal trophic mode (Hobbie et al., 2001). As mycorrhizal fungi promote secondary metabolite production in medicinal plants (Zhao Y. et al., 2022), the positive correlation between *Aleuria* and atractylon content may indicate an underlying ecological link, although the specific mechanism remains unclear. In contrast, the characterized species of *Volutella* are functionally biased toward plant pathogenicity. For example, *V. buxi* causes Boxwood Volutella stem blight or canker, and *V. pachysandricola* causes Volutella blight in Japanese pachysandra (*Pachysandra terminalis*; Babu et al., 2015). The increased relative abundance of the potentially pathogenic taxon *Volutella* may increase the risk of disease stress in *A. chinensis* and be associated with changes in plant resource allocation and secondary metabolism, thereby providing a possible ecological explanation for the observed negative correlation. These findings suggest that continuous cropping results in a decline in the yield and medicinal quality of *A. chinensis* in the Horqin Sandy Land and that this decline is associated with imbalances in the rhizosphere soil chemical properties and alterations in specific microbial taxa.

This study has some limitations. First, the contents of bioactive compounds in *A. chinensis* and soil chemical variables were measured at four developmental stages, whereas rhizosphere microbial sequencing was performed only at the harvest stage. Therefore, the temporal sequence of microbial changes and changes in plant yield and metabolite accumulation could not be determined, thereby complicating the inference of causal relationships between them. In addition, three independent field plots were established for each treatment. The limited number of independent field replicates may compromise statistical power, particularly in microbiome and correlation analyses, and may undermine the robustness of LEfSe-based biomarker identification, potentially rendering it sensitive to outliers in individual plots. Therefore, the biomarkers, correlations, and functional results reported in this study should be considered exploratory. Future studies should incorporate longitudinal microbiome sampling across different developmental stages and include an increased number of independent field plots to further validate these findings.

## 5 Conclusion

This study demonstrated that continuous cropping significantly reduced the yield of *A. chinensis* and the accumulation of its major bioactive compounds, including atractylodin, atractylon, and β-eudesmol, in the Horqin Sandy Land. Continuous cropping substantially altered the chemical properties and relative abundance of specific microbial taxa in rhizosphere soils. Continuous cropping increased the soil pH, EC, and AK, whereas significantly decreasing AN and OM contents. At the microbial level, continuous cropping promoted the enrichment of *Fusarium* and *Volutella*, while reducing the relative abundance of *Nocardioides* and *Arthrobacter*. The predicted microbial functional potential associated with carbon and nitrogen cycling significantly decreased under continuous cropping. Correlation analysis further showed that *A. chinensis* yield was negatively correlated with EC and positively correlated with OM and the relative abundances of *Heydenia* and *Enterocarpus*. The atractylon content was positively correlated with the relative abundances of *Aleuria* and *Acremonium*, and negatively correlated with the relative abundance of *Volutella*. Overall, these findings indicate that continuous cropping reduces the yield and content of bioactive compounds in *A. chinensis*, concurrent with alterations in the chemical properties of the rhizosphere soil and the relative abundance of specific microbial taxa. Additionally, a decline in yield and medicinal quality was associated with changes in certain soil chemical properties and key microbial taxa. These findings provide new insights into CCOs and their ecological responses in medicinal plants and provide a theoretical foundation for developing sustainable cultivation and management strategies for medicinal plants in Horqin Sandy Land.

## Data Availability

The datasets presented in this study can be found in online repositories. The names of the repository/repositories and accession number(s) can be found at: https://nmdc.cn/resource/genomics/sra/detail/NMDC40126860, NMDC40126860; https://nmdc.cn/resource/genomics/sra/detail/NMDC40126861, NMDC40126861; https://nmdc.cn/resource/genomics/sra/detail/NMDC40126862, NMDC40126862; https://nmdc.cn/resource/genomics/sra/detail/NMDC40126863, NMDC40126863; https://nmdc.cn/resource/genomics/sra/detail/NMDC40126864, NMDC40126864; https://nmdc.cn/resource/genomics/sra/detail/NMDC40126865, NMDC40126865; https://nmdc.cn/resource/genomics/sra/detail/NMDC40126866, NMDC40126866; https://nmdc.cn/resource/genomics/sra/detail/NMDC40126867, NMDC40126867; https://nmdc.cn/resource/genomics/sra/detail/NMDC40126868, NMDC40126868; https://nmdc.cn/resource/genomics/sra/detail/NMDC40126869, NMDC40126869; https://nmdc.cn/resource/genomics/sra/detail/NMDC40126870, NMDC40126870; and https://nmdc.cn/resource/genomics/sra/detail/NMDC40126871, NMDC40126871.

## Footnotes

1
https://www.omicstudio.cn/

2
https://www.bioincloud.tech/
