# Supplementary material for: Effects of continuous cropping on yield, medicinal quality, and rhizosphere soil microecological environment of Atractylodes chinensis in the Horqin Sandy land
Source: Front Microbiol. 2026 Oct 27;17:1943837. doi: 10.3389/fmicb.2026.1943837 (PMC13748433; doi:10.3389/fmicb.2026.1943837)
Supplement: Supplementary file 1 [file Supplementary_file_1.DOCX]

| Soil  variable  Cropping  systems | | pH | EC  (μS/cm) | AN  （mg/kg） | AP  （mg/kg） | AK  （mg/kg） | OM  （g/kg） |
| --- | --- | --- | --- | --- | --- | --- | --- |
| T1 | 8.30±0.02a | | 57.93±0.35a | 56.47±2.65a | 30.98±1.52a | 51.40±4.15a | 24.81±1.55a |
| T2 | 8.29±0.04a | | 58.47±1.16a | 55.77±3.86a | 30.90±1.27a | 51.42±4.16a | 24.51±1.83a |

Supplementary Table S1. Baseline soil chemical properties of the experimental plots before planting. T1, continuous cropping; T2, crop rotation. EC, electrical conductivity; AN, alkaline hydrolysis nitrogen; AP, available phosphorus; AK, available potassium; OM, organic matter. Data are presented as means ± SD (n = 3). Differences between T1 and T2 were analyzed using independent-samples *t*-tests, and no significant differences were detected between the two treatments (*P* > 0.05).

Supplementary Figure S1. Effects of continuous cropping on rhizosphere microbial β-diversity of *A.chinensis*. (A) bacterial communities; (B) fungal communities. T1, continuous cropping; T2, crop rotation.
